# Selenium Nanoparticles Attenuate Oxidative Stress and Testicular Damage in Streptozotocin-Induced Diabetic Rats

**DOI:** 10.3390/molecules21111517

**Published:** 2016-11-19

**Authors:** Mohamed A. Dkhil, Rafat Zrieq, Saleh Al-Quraishy, Ahmed E. Abdel Moneim

**Affiliations:** 1 Department of Zoology, College of Science, King Saud University, Riyadh 11451, Saudi Arabia; mohameddkhil@yahoo.com (M.A.D.); guraishi@yahoo.com (S.A.-Q.); 2 Department of Zoology and Entomology, Faculty of Science, Helwan University, Cairo 11795, Egypt; 3 Department of Clinical Laboratory Sciences, College of Applied Medical Sciences, University of Hail, Hail 2440, Saudi Arabia; rafat.zrieq@uni-duesseldorf.de

**Keywords:** selenium, nanoparticles, diabetes, oxidative stress, PCNA, apoptosis, rats

## Abstract

We investigated the protective and antioxidative effects of selenium nanoparticles (SeNPs) in streptozotocin STZ-induced diabetic rats. STZ-diabetic rats were exposed daily to treatments with SeNPs and/or insulin and then the effect of these treatments on the parameters correlated to oxidative damage of the rat testes were assessed. Biochemical analysis revealed that SeNPs are able to ameliorate the reduction in the serum testosterone caused by STZ-induced diabetes. Furthermore, SeNPs could significantly decrease testicular tissue oxidative stress markers, namely lipid peroxidation and nitric oxide. In contrast, treatment of the STZ-diabetic rats with SeNPs increased the glutathione content and antioxidant enzyme activities in testicular tissues. Moreover, microscopic analysis proved that SeNPs are able to prevent histological damage in the testes of STZ-diabetic rats. Molecular analysis revealed that the mRNA level of *Bcl-2* (B-cell lymphoma 2) is significantly upregulated. On the contrary, the mRNA level of *Bax* (*Bcl-2* Associated X Protein) was significantly downregulated. Furthermore, treatment of STZ-diabetic rats with SeNPs led to an elevation in the expression of PCNA (Proliferating Cell Nuclear Antigen Gene). Interestingly, the insulin treatment also exhibited a significant improvement in the testicular function in STZ-diabetic rats. Collectively, our results demonstrated the possible effects of SeNPs in attenuating diabetes-induced oxidative damage, in particular in testicular tissue.

## 1. Introduction

Impotence and impaired spermatogenesis are consequences of diabetes, and it is reported that fertility dysfunction is a common complication in diabetic patients. The underlying mechanisms for this are, however, still not well understood [1]. The most frequent detrimental effects of diabetes on the male reproductive system include a decrease in the weight of body and reproductive organs, lower levels of testosterone, disruption of the spermatogenesis, lower sperm counts with high mortality and alterations in the morphology of the components of testicular tissue [2,3].

Previous reports have revealed that both oxidative stress and alterations in the antioxidant enzyme system are the two most important factors leading to reproductive and sexual dysfunction. In this context, Gumieniczek et al. [4] and Shrilatha and Muralidhara [5] found that oxidative stress resulting in lipid and protein peroxidation was evident 14 days after the commencement of diabetes.

The importance of selenium (Se) for successful reproduction has long been recognized in animal husbandry [6]. It is important in male reproduction due to its role in testosterone biosynthesis and subsequently in the formation and typical development of spermatozoa [7]. Thus, Se is a key factor in spermatogenesis and male fertility [8]. Se-regulated spermatogenesis is mediated by two selenoproteins. The first of these is the phospholipid hydroperoxide glutathione peroxidase (PHGPx/GPx4), the most abundant selenoprotein in germ cells in the testes and considered to be important for male fertility. The second is selenoprotein P, which serves as a source of selenium in the testes [9]. Furthermore, there have been many studies into how Se mitigatestoxicant-induced testicular oxidative injury by regulating the activities of antioxidant enzymes.

Recently, elemental Selenium (Se) nanoparticles (SeNPs) have become a point of interest in nanotechnology treatment due to their unique biological activities and low toxicity [10]. Thus, the goal of this study was to clarify the potential role of the administration of SeNPs on diabetes-related lesions, in particular in respect to the oxidative damage in the testicular tissue of streptozotocin-diabetic rats.

## 2. Results

**Figure 1:**
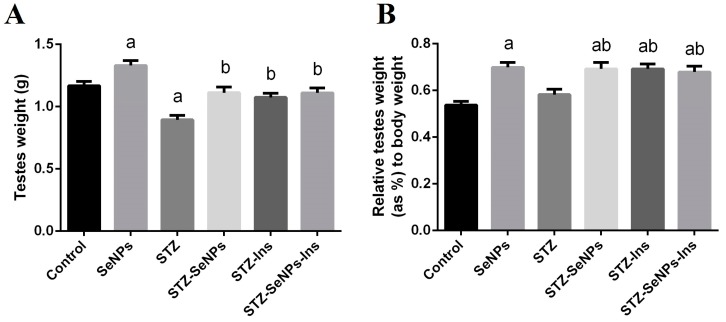
Impact of SeNPs and/or insulin on (**A**) the absolute and (**B**) relative weight of the testis in control and STZ-diabetic rats. The weight of rat testis and total body weights were measured over 28 days. Values are mean ± SEM (*n* = 7). ^a^ *p* < 0.05, significant change with respect to Control group; ^b^ *p* < 0.05, significant change with respect to Diabetic group.

Although STZ-diabetic rates showed a significant reduction in the weight of their testes compared to control rats (Figure 1, left graph), the weight of the testes relative to the overall weight of STZ-diabetic rats was not affected (Figure 1, right graph), suggesting that this reduction is due to a general loss of eight among the STZ-diabetic rats. Interestingly, four weeks of SeNPs and/or insulin treatment had a significant (*p* < 0.05) effect in terms of preventing this reduction in testis weight and in fact increased the relative testis weight compared to the diabetic rats. SeNPs also per se significantly increased both the absolute and relative weight of the testes compared with that in the control rats. Thus, STZ reduces rat body weights including testes, and, like insulin, SeNPs increase the weight of the testes in rats even in those affected due to diabetes.

**Figure 2:**
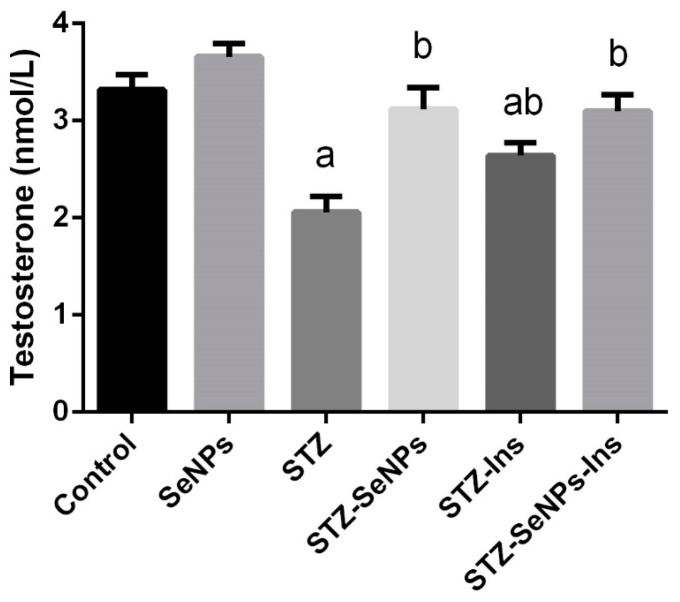
Impact of SeNPs and/or insulin on the serum testosterone levels in control and STZ-diabetic rats. Values are mean ± SEM (*n* = 7). ^a^ *p* < 0.05, significant change with respect to Control group; ^b^ *p* < 0.05, significant change with respect to Diabetic group.

Serum levels of testosterone showed a significant (*p* < 0.05) reduction in diabetic rats compared to the control rats. Interestingly, SeNPs non-significantly increased the serum testosterone levels in non-diabetic rats, suggesting that SeNPs have a role in the synthesis of testosterone (Figure 2). Furthermore, four weeks of SeNPs and/or insulin administration could significantly recover the serum testosterone to the normal levels expected in non-diabetic rats (Figure 2).

**Figure 3:**
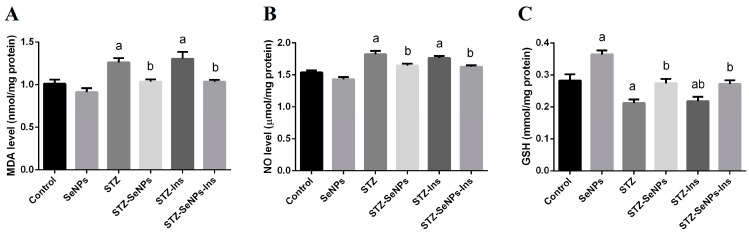
Impact of SeNPs and insulin on oxidative stress markers [(**A**) lipid peroxidation: LPO, indicated by MDA; (**B**) nitric oxide: NO and (**C**) glutathione: GSH] in the testis of control and STZ-diabetic rats. Values are mean ± SEM (*n* = 7). ^a^ *p* < 0.05, significant change with respect to Control group; ^b^ *p* < 0.05, significant change with respect to Diabetic group.

STZ-diabetic rats showed an elevation in malondialdehyde (MDA) and nitric oxide (NO) levels compared to the control animals (Figure 3, left and middle graphs, STZ compared to control). Thus, STZ increased the level of MDA and NO while SeNPs reduced these levels. Interestingly, in STZ-diabetic/SeNP treated rats, these markers were found to be close or similar to the normal values present in control rats. (Figure 3, left and middle graph, SeNPs compared to STZ and control). Insulin treatment failed to prevent oxidative stress in the testis with outcomes being similar to those in untreated diabetes (Figure 3, left and middle graph, Ins compared to STZ and control). Additionally, STZ-induced diabetes significantly decreased GSH levels (*p* < 0.05) in testicular tissue (Figure 3, right graph, STZ compared to control). This reduction in GSH contents was significantly attenuated by treatment with SeNPs and/or insulin (Figure 3, right graph, SeNPs and/or insulin compared to STZ).

**Figure 4:**
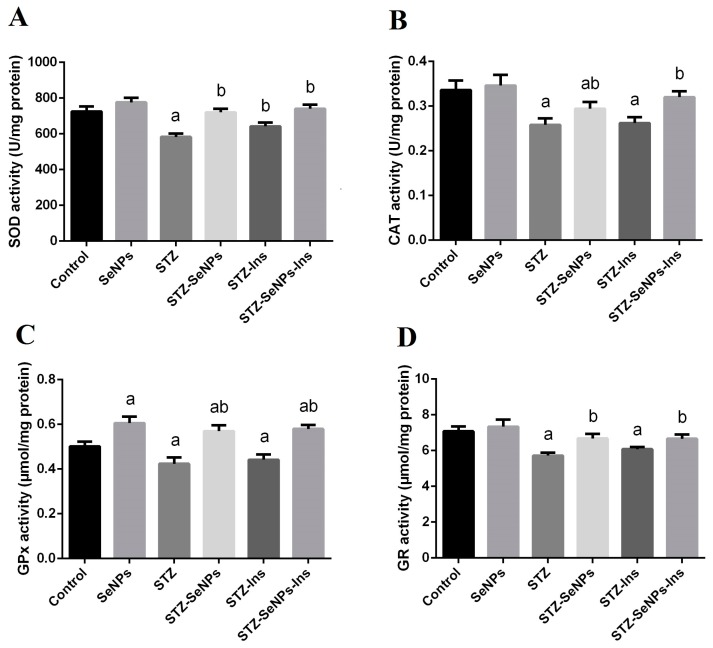
Effect of SeNPs and insulin on antioxidant enzyme activities [(**A**) superoxide dismutase: SOD; (**B**) catalase: CAT; (**C**) glutathione peroxidase: GPx and (**D**) glutathione reductase: GR] in the testis of control and STZ-diabetic rats. Values are mean ± SEM (*n* = 7). ^a^ *p* < 0.05, significant change with respect to Control group; ^b^ *p* < 0.05, significant change with respect to Diabetic group.

**Figure 5:**
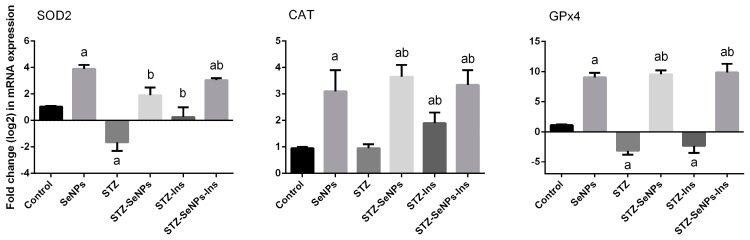
Impact of SeNPs and/or insulin on gene expression of antioxidant enzymes in the testis of control and STZ-diabetic rats. Results (mean ± SEM of the triplicate) were settled according to the mRNA level of *β-actin* and are shown as fold induction (in log2 scale) of the mRNA level in the control. *SOD2*: superoxide dismutase isoform 2; CAT: catalase and GPx4: phospholipid hydroperoxide glutathione peroxidase.

To study the influence of diabetes in respect to oxidative damage to the testes, the action of the antioxidant defence system was assessed by estimating the activity of SOD, CAT, GPx and GR enzymes. The induction of diabetes resulted in a significant reduction in the activities of all of these enzymes (Figure 4, comparing STZ to the control in all graphs). In contrast, treatment of non-diabetic rats with SeNPs increased the activity of GPx compared to the untreated diabetic rats (Figure 4, comparing SeNPs to control in all graphs). Interestingly, STZ-diabetic rats treated with SeNPs exhibited increased activities of all the enzymes tested (Figure 4, comparing STZ-SeNPs to STZ in all graphs). Thus, SeNPs seemed to help recover the impaired activity of antioxidant enzymes in diabetic rats. Our qPCR results showed that the expression of *SOD2* and *GPx4* genes was downregulated in the testis of STZ-diabetic rats (Figure 5, left and right graphs, STZ compared to control). In contrast, treatment of normal non-diabetic rats with SeNPs resulted in an upregulation of *SOD2* and *GPx4* genes (Figure 5, left and right graphs, SeNPs compared to the control). As expected, treatment of diabetic rats with SeNPs also increased the gene expression of both *SOD2* and *GPx4* (Figure 5, left and right graphs, STZ-SeNPs compared to STZ). The expression of *CAT* did not seem to be affected in STZ-diabetic rats (Figure 5, middle graph, STZ compared to control). Treatment of both diabetic and non-diabetic rats, however, could elevate the expression of *CAT* (Figure 5, middle graph, SeNPs compared to STZ and the control).

**Figure 6:**
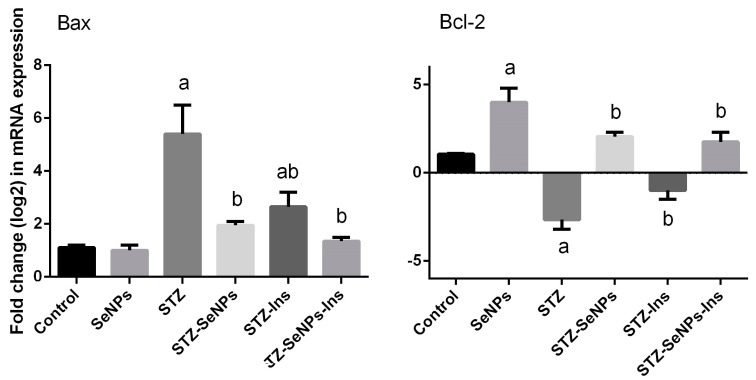
Impact of SeNPs and/or insulin on the expression of apoptosis genes in the testis in control and STZ-diabetic rats. Results (mean ± SEM of triplicate) were arrived according to the mRNA level of *β-actin* RNA and are shown as fold induction (in log2 scale) of the mRNA level in the control.

Diabetic rats showed significant upregulation in *Bax* expression compared to the control rats (Figure 6), but the level of *Bcl-2* was found to be lower in the testicular tissue of diabetic rats than the control group. Nevertheless, treatment with SeNPs significantly (*p* < 0.05) prevented the changes in *Bax* and *Bcl-2* mRNAs observed in the diabetic rats. Moreover, the effect of SeNPs was much more pronounced than that of the standard drug, insulin.

**Figure 7:**
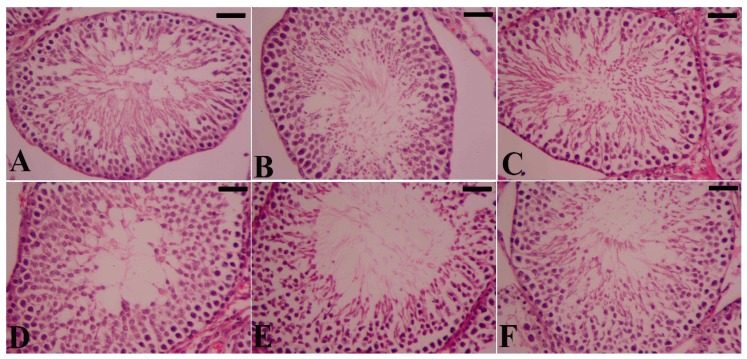
Impact of SeNPs and/or insulin on the histology of the testis in diabetic rats. (**A**) control rats; showing typical testicular architecture; (**B**) SeNPs-treated rats; showing typical spermatogenic cells in the seminiferous tubules; (**C**) STZ-diabetic rats; showing severe testicular damage; (**D**–**F**) STZ-SeNPs-treated group, STZ-Ins-treated rats and STZ-SeNPs-Ins-treated rats, respectively; SeNPs and/or insulin ameliorated the defects of the spermatogenic cells in the seminiferous tubules caused by diabetes. Tissues were stained with haematoxylin and eosin. Scale bar = 50 μm.

Diabetic rats showed that a high level of blood glucose (≥298 mg/dL) caused alterations in the normal histological architecture of the testes compared to the control rats (Figure 7, compare C to A). In particular, the layers of the epithelium were severely impaired varying from totally to partially disorganized and also with obviously impaired organization of the stages of spermatogenesis. In addition, diabetic rats exhibited atrophy in the seminiferous tubules with arrested spermatogenesis and necrotic cells evident in the luminal compartment associated with a lack of spermatogonia in the basal portion, in particular in the heavily damaged tubules. In contrast, treatment with SeNPs appeared to restore the normal structure (Figure 7, compare D to C). Histological staining of the testis in diabetic rats showed a considerable number of thickened seminiferous tubules that extended to the basement membrane. Those diabetic rats treated with SeNPs, however, regained normal seminiferous tubules lined by several layers of spermatogenic cell series, similar to those of the control (Figure 7D).

**Figure 8:**
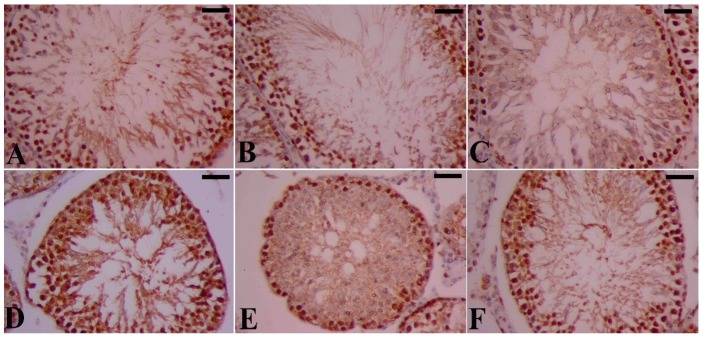
PCNA staining of the testis of STZ-diabetic rats. Spermatogenic cells from the rat testis were stained with a specific antibody against PCNA. (**A**) control rats; showing the typical presence of spermatogenic cells in the seminiferous tubules; (**B**) SeNPs-treated rats; showing an abundance of spermatogenic cells in the seminiferous tubules; (**C**) STZ-diabetic rats, showing weaker PCNA-immunoreactivity and fewer spermatogenic cells in the seminiferous tubules compared to A and B; (**D**) STZ-SeNPs-treated rats: a stronger PCNA-immunoreactivity with an increased number of spermatogenic cells in the seminiferous tubules was observed compared to C; (**E**) STZ-Ins-treated rats, fewer positive nuclei for PCNA in the spermatogenic epithelium cell were observed; and (**F**) STZ-SeNPs-Ins-treated rats, a marked elevation in the expression of PCNA was seen, as indicated by the increased PCNA-immunoreactivity compared to C and E. Scale bar = 50 μm.

Figure 8 illustrates, for all groups, the cells that were immunoreactive positive for the Proliferating Cell Nuclear Antigen Gene (PCNA), once again showing that both the seminiferous tubules and interstitial tissue compartments, were normally structured in the control and SeNP groups. Although spermatogonia and primary spermatocytes mainly contained PCNA immunoreactive cells, secondary spermatocytes or spermatids appeared to have lower contents of PCNA. It is clear from Figure 8C that the number of PCNA-positive germinal cells was markedly reduced in STZ-diabetic rats compared to control rats, and that PCNA staining was likely restricted to spermatogonia and primary spermatocytes with no PCNA content either in the secondary spermatocytes or spermatids. Finally, SeNPs treated diabetic rats exhibited plenty of PCNA immunostained cells in the tissues of the testis, indicating high expression of PCNA (Figure 8, comparing D to C). The diabetic rats that were treated with insulin alone, however, showed fewer positive nuclei for PCNA in the spermatogenic epithelium cells (Figure 8, comparing E to C).

## 3. Discussion

Diabetes has a deleterious effect on the reproductive system of males. Lesions in the reproductive system of rats exposed to STZ are not caused directly by the toxicity of STZ or any of its by-products, but due to the induction of diabetes [11]. Increasing evidence indicates that hyperglycaemia could induce oxidative stress as well as apoptosis by inducing the formation of ROS and inhibiting the efficiency of the antioxidant defence system. ROS occur very early during diabetes development and play a major role in its complications, including male infertility [12].

We observed that diabetes induction in rats could significantly induce oxidative stress in the testis by increasing the levels of LPO and NO and decreasing the level of GSH. Furthermore, induction of diabetes using STZ in rats attenuated the activities of the antioxidant enzymes in the testicular tissues including SOD, CAT, GR and GPx. These effects were accompanied by the induction of apoptosis, as indicated by the upregulation of the pro-apoptotic gene (*Bax*) and the downregulation of the anti-apoptotic gene (*Bcl-2*). In the context, our data suggests that SeNPs are a potent agent for the prevention and treatment of diabetes-induced reproductive impairment.

STZ-induced diabetes is associated with body weight loss, which is combined with organ weight loss. Our data in Figure 1 show a significant reduction in the weight of the testis in STZ-diabetic rats compared to the control group, suggesting that the testicular weight loss may be one of the reasons to cause its dysfunction. Diabetes-induced weight loss of the male reproductive organs has been reported to be induced by oxidative stress and apoptosis, leading to atrophy of the sex organs [11].

In the present study, we found that induction of diabetes in rats resulted in reduction of Sertoli and spermatogenic cells and is associated with degeneration in the spermatogenic cell series and thickened interstitial vessel walls. Diabetes has been reported to reduce the number of spermatogenic cells, and the diameter of the seminiferous tubules, as documented by Guneli et al. [13], and to induce cell apoptosis and atrophy in the seminiferous tubules, which are both indicators for the failure of spermatogenesis [14]. In contrast, we demonstrated that SeNPs prevent and minimize diabetes-induced spermatogenic cell loss in the diabetic rats. These observations indicate that the spermatogenic cell-protective effects of Se may reflect the antioxidant nature of selenium in biological systems as well as its fundamental role in sperm maturation [15].

Regarding the effect of diabetes on male fertility, 20% to 64% of diabetic men are suffering from hypogonadism [16]. Our results likewise showed a lower testosterone level in diabetic rats. During diabetes, reduction of testosterone levels might be due to changes in the body composition, androgen receptor polymorphisms, glucose transport and decreased level of antioxidants [11,16]. Indeed, a direct relationship between insulin, testosterone and gonadotropins has been reported. Reduction of insulin inhibits the secretion of the gonadotropins, follicle-stimulating hormone (FSH) and luteinizing hormone (LH). Both hormones stimulate the production of androgens including testosterone. Thus, a low level of gonadotropins reduces the production of testosterone in the testicular tissue [17,18]. Testosterone is required for spermatogenesis and the activities of Sertoli cells. Fortunately, SeNPs significantly elevated testosterone level in STZ-diabetic rats compared with non-treated STZ-diabetic rats. Behne et al. [7] reported that selenium stimulates LH and positively affects the biosynthesis of testosterone. In fact, Se is an important trace element that is crucial for male fertility and plays a role in sperm formation and development [19].

Hyperglycaemia induces oxidative stress in the testis by increasing the formation of free radicals and reducing the activities of endogenous antioxidant enzymes, subsequently leading to oxidative stress [3]. Increased production of ROS causes mitochondrial injury and lipid peroxidation in both, germ and Leydig cells and, subsequently, a defect in sperm production [20]. Our findings, revealed that lipid peroxidation is significantly increased in the testis of STZ-diabetic rats, in agreement with other studies [3,20]. Saravanan and Pari [21], for example, reported that, hypoinsulinemia stimulates the activity of fatty acyl coenzymes A oxidase, which oxidizes fatty acids and therefore results in lipid peroxidation. Lipid peroxidation leads to decrease membrane fluidity and disturbs the interaction between the membrane-bound enzymes and receptors, ultimately resulting in cell damage.

We also observed that the activities of testicular GPx, CAT and SOD were significantly inhibited in STZ-diabetic rats. These results clearly suggest that increased oxidative stress might be due to reduced activities of testicular antioxidant enzymes [22,23]. They also show, however, that SeNPs were able to restore the activity of these antioxidant enzymes and reduce the levels of lipid peroxidation and nitric oxide.

In the present study, we expected that insulin treatment could prevent oxidative stress in the testicular tissue. However, diabetic rats treated with insulin showed similar oxidative stress to untreated diabetic rats, as examined by measuring LPO, NO and GSH levels, these findings were unexpected. It is perhaps that the tight blood glucose level control using intensive insulin monotherapy will be able to prevent oxidative stress.

At the molecular level, we found that, *Bax* expression was substantially elevated in the testicular tissue of STZ-diabetic rats, while *Bcl-2* was significantly downregulated. Furthermore, the *Bax/Bcl-2* ratio, a main index for apoptosis, was significantly elevated; indicating hyperglycaemia-induced apoptosis in the testis of STZ-diabetic rats. This may be mediated by the mitochondrial pathway. Hyperglycaemia increases the expression of Bax protein and causes apoptosis in PC12 cells [24]. The association of apoptosis with down-regulation in *Bcl-2* expression has been observed in the testes of diabetic rats [2,25]. Our study revealed that treatment of STZ-diabetic rats with SeNPs downregulated the expression of *Bax* and upregulated the expression of *Bcl-2*. These results suggested that Se inhibits apoptosis via its ability to modulate *Bax/Bcl-2* expressions, decreasing the ratio of *Bax/Bcl-2*. This, in turn, provides evidence for the anti-apoptotic properties of selenium. In fact, Faucher et al. reported that GPx1 conserves cells against apoptotic/oxidant molecules in two different ways: (i) GPx1 can scavenge the physiological H_2_O_2_ pool, and therefore loses its regulatory role on the transcription of its target genes including *Bax*; or (ii) alternately, GPx1 could influence *Bax* expression in an unknown way [26].

Proliferating cell nuclear antigen is an intranuclear polypeptide and a cofactor of DNA polymerase delta that is essential for replication, excision and repair [27]. Since spermatogenesis is a complicated cell cycle of fast proliferating cells ending with the formation of sperms, we used PCNA in this study as a tool to assess spermatogenesis. We observed that PCNA positive cells were strongly expressed in spermatogonia and early-stage spermatocytes of non-treated control rats. In contrast, the density of PCNA positive testicular germ cells was significantly diminished in STZ-diabetic rats, which is an indication of disruption in proliferation and spermatogenesis. Previous studies have demonstrated that the increase in PCNA expression in testicular tissue is an indication of high proliferative activity and stimulation of spermatogenesis, and this expression is downregulated in diabetes [27,28]. Our study proved that the negative effect of lowered PCNA expression in STZ-diabetic rats is recovered by treatment with SeNPs. Thus, the upregulation of PCNA led to the promotion of cell cycle progression and the reduction of apoptosis. Se compounds are implicated in modulating a variety of cellular activities leading to cell proliferation and survival [29].

## 4. Experimental Section

### 4.1. Preparation and Characterization of Selenium Nanoparticles

Se nanoparticles were supplied by Prof. Dr. Xueyun Gao (the Institute of High Energy Physics, Beijing, China). To synthesis SeNPs, sodium selenite was reduced with glutathione in the presence of bovine serum albumin (BSA) [30]. The suspension of red amorphous SeNPs was collected. The size and shape of the SeNPs were imaged by means of transmission electron microscopy (TEM) using a TEM Philips CM100 instrument (FEI, Eindhoven, The Netherlands) at 80 kV accelerating voltage. The diameter of the SeNPs collected from the target fraction was in the range of 10 to 80 nm [10].

### 4.2. Animals

Adult male Wistar rats (110–140 g, two-month old and fed ad libitum) were used in this study. The rats were purchased from the Holding Company for Biological Products and Vaccines (VACSERA, Cairo, Egypt) and fed ad libitum. All animal studies were performed according to the standards of animal care set out in Egyptian rules and the National Institutes of Health (NIH) Guidelines for the Care and Use of Laboratory Animals, 8th edition. Diabetes was induced by injecting the rat intraperitoneally (i.p.) with 55 mg/kg body wt streptozotocin (STZ; Sigma, St. Louis, MO, USA). The STZ, diluted in a 0.05 M citrate buffer (pH 4.5), was freshly prepared immediately before injection. To monitor blood glucose level in STZ-diabetic rats, blood drops from the rats were taken every three days and tested by using an Accu-check blood glucose meter (Roche Diagnostics, Basel, Switzerland). STZ-diabetic rats showing blood glucose levels ≥200 mg/dL (15 mM) seven days after the STZ injection were considered hyperglycaemic. In the control group, the same type of rats were injected with a citrate buffer only.

### 4.3. Experimental Design

Verified STZ-diabetic rats were divided into four groups, with seven rats in each. Control rats (i.e., those injected with a citrate buffer only) were divided into two groups, again with seven rats in each group.

The first group of STZ-diabetic rats received a physiological NaCl-solution (STZ group). In the second group, STZ-diabetic rats were treated once a day with 0.1 mg of SeNPs/kg of rat weight (STZ-SeNPs group). The third group, STZ-diabetic rats, was treated with daily 6 U of standard drug insulin/kg of the rat weight (STZ-Ins) (Ins; 6 U/kg, subcutaneous administration; s.c.). In the fourth group of STZ-diabetic rats, the animals were treated with both SeNPs and insulin at the concentration indicated above (STZ-SeNPs-Ins). Additionally, the two control groups (the 14 rats injected with citrate buffer) were injected either with a physiological NaCl-solution (control group) or with 0.1 mg/kg (SeNPs group). All buffer and SeNP treatments were administered orally [30], while insulin treatment was administered subcutaneously daily for a period of 28 days. The rats were then killed by mild ether anaesthesia and the testes were removed. Additionally, the rat serum was isolated out of blood collected from the abdominal aorta, using a syringe puncture. The obtained testes and serum were used for further histological, molecular and/or biochemical studies. The weight of all the animals was measured prior to the different types of treatment and again prior to sacrifice.

### 4.4. Changes in the Testes Index of Rats

The relative weight of testes was calculated according to the weight of the left testis (LT) as follows: LT/body weight × 100.

### 4.5. Estimation of Serum Testosterone

The serum testosterone hormone of the rats was quantitatively measured by an enzyme linked immunosorbent assay (ELISA) using a specific kit for quantifying testosterone in the serum isolated from rat blood (BioVendor, Gunma, Japan). The experiment was performed as instructed by the manufacturer.

### 4.6. Preparation of Testis Homogenates

The testes were homogenized in ten volumes of ice-cold medium of 50 mM Tris-HCl (pH 7.4). Testis homogenates were centrifuged at 1000× *g* for 10 min at 4 °C. The supernatants were used for the investigations of oxidative stress and antioxidant enzyme activities. The protein level of the homogenates was assessed using the method of Lowry et al. [31].

### 4.7. Oxidative Stress of Testes

To determine the lipid peroxidation (LPO) in the testis, the testis homogenate was incubated with 1 mL of 10% trichloroacetic acid and 1 mL of 0.67% thiobarbituric acid at 100 °C for 30 min [32]. In the presence of malondialdehyde (MDA), which reacts with trichloroacetic acid, the colored complex formed with a maximum absorbance of 535 nm expressed the amount of MDA. Meanwhile, the nitric oxide level in the testis homogenate was determined by the optimized acid reduction method as described by Green et al. [33]. The testicular glutathione (GSH) was determined by the reduction of Elman’s reagent as has been described previously [34].

### 4.8. Antioxidant Status

The activities of several antioxidant enzymes were determined as indicators for the assessment of oxidative stress in the testes. First, superoxide dismutase (SOD) was determined based on its ability to inhibit nitroblue tetrazolium (NBT), as described in Peltola et al. [35]. Second, the activity of testicular catalase (CAT), which is essential for removing the hydrogen peroxide produced by SOD, was also determined by mixing 50 μL of testis homogenate to 30 mM H_2_O_2_ in 50 mM of potassium phosphate buffer (pH 7.8). The consumption of hydrogen peroxide was quantified by a photometer at 340 nm for 120 s at 20 s intervals Beers and Sizer [36]. The activity of SOD and CAT was expressed as units/mg proteins. Third, glutathione reductase (GR) was assayed indirectly based on the oxidation of NADPH to NADP^+^. The decrease in absorbance of NADPH at 340 nm expressed H_2_O_2_ reduction by glutathione peroxidase (GPx) to alcohol. Finally, GPx activity in the testis homogenates was also indirectly examined by the assay described by Paglia and Valentine [37]. In this assay, oxidized glutathione (GSSG) produced by GPx activity is regulated by the excess GR in the assay. The GR activity was monitored by following the loss of NADPH (at 340 nm).

### 4.9. Quantitative Real-Time PCR

Total RNA of the testicular tissues was isolated using an RNeasy Plus Minikit (Qiagen, Valencia, CA, USA). In addition, 1 μg of the isolated RNA was used as a template together with random primers to synthesize cDNA using the RevertAid™ H Minus Reverse Transcriptase (Fermentas, Thermo Fisher Scientific Inc., Waltham, MA, USA). Each cDNA sample was run in triplicate for real-time PCR analysis. *β-Actin* (accession number: NM_031144.3; sense: 5′-GGCATCCTGACCCTGAAGTA-3′; antisense: 5′-GGGGTGTTGAAGGTCTCAAA-3′) served as a house keeping gene. Real-time PCR reactions were performed using Power SYBR^®^ Green (Thermo Fisher Scientific Inc., Waltham, MA, USA) on the Applied Biosystems 7500 system. The typical thermal profile for the PCR reaction was 95 °C for 4 min, followed by 40 cycles of 94 °C for 60 s and 55 °C for 60 s. For relative quantitation of gene expression, the log2 of 2^−ΔΔCt^ was used based on the method of Pfaffl [38], where ΔΔCt was calculated by subtracting the *β-actin* cycle threshold value (Ct) from each of the target gene Ct. The ΔΔCt value was then calculated by subtracting the resultant ΔΔCt values from the mean ΔΔCt value of the control. The PCR primers for the following genes were synthesized by Jena Bioscience GmbH (Jena, Germany): *SOD2* (accession number: NM_001270850.1; sense: 5′-AGCTGCACCACAGCAAGCAC-3′; antisense: 5′-TCCACCACCCTTAGGGCTCA-3′), *CAT* (accession number: NM_012520.2; sense: 5′-TCCGGGATCTTTTTAACGCCATTG-3′; antisense: 5′-TCGAGCACGGTAGGGACAGTTCAC-3′), *GPx4* (accession number: NM_001039849.2; sense: 5′-ATTCCCGAGCCTTTCAACCC-3′; antisense: 5′-TATCGGGCATGCAGATCGAC-3′), *Bcl-2* (accession number: NM_016993.1; sense: 5′-CTGGTGGACAACATCGCTCTG-3′; antisense: 5′-GGTCTGCTGACCTCACTTGTG-3′) and *Bax* (accession number: NM_017059.2; sense: 5′-GGCGAATTGGCGATGAACTG-3′; antisense: 5′-ATGGTTCTGATCAGCTCGGG-3′). Primers were designed using the web service of Primer-Blast from NCBI (https://www.ncbi.nlm.nih.gov/tools/primer-blast/index.cgi?LINK_LOC=BlastHome).

### 4.10. Histological Changes

Testis tissues were fixed using neutral buffered formalin (10%) for 24 h and dehydrated with ethyl alcohol. After that, the fixed tissues were cleared by xylene and subsequently mounted in molten paraplast. Sections were managed in a range of 4–5 μm. The tissue sections were then stained with haematoxylin-eosin in order to visualize seminiferous tubules and spermatogenic cells using a Nikon microscope (Eclipse E200-LED, Tokyo, Japan).

### 4.11. Immunohistochemistry

The peroxidase/anti-peroxidase (PAP) method was used to assess the immunocytochemical reactions in the testis [39]. Formaldehyde-fixed, paraffin-embedded testicular tissue sections were used. To reduce non-specific peroxidase reactions and background, tissue sections were incubated with methanol containing 3% H_2_O_2_ and normal goat serum for 30 min. Subsequently, the tissue sections were incubated with a specific first antibody that recognized the proliferative cell nuclear antigen (PCNA) (Santa Cruz Biotechnology, Dallas, Texas, USA, dilution, 1:2000) and subsequently with HRP-conjugated goat anti-mouse IgG as a secondary antibody (1:5000; Sigma, St. Louis, MO, USA). After that, tissue sections were incubated with the PAP complex (dilution, 1:200) for 90 min. Diaminobenzidine was used as chromogen. Finally, the samples were counterstained with haematoxylin and analyzed using a light microscope.

### 4.12. Statistical Analysis

Values are expressed as the mean ± standard error of the mean (SEM). Data were statistical analyzed by one-way analysis of variance (ANOVA). For the comparison of significance between groups, Duncan’s test was used as a post hoc test according to the Statistical Package for the Social Sciences (SPSS version 20.0, Chicago, IL, USA). A *p*-value < 0.05 was considered statistically significant.

## 5. Conclusions

In conclusion, the present study demonstrated that diabetes induces severe biochemical and histopathological changes in the testes of STZ-diabetic rats. Selenium nanoparticles serve significantly to ameliorate these diabetes-caused testicular dysfunctions, in particular by decreasing oxidative stress and apoptosis.
